# CSF Proteome Analysis of *p*‐Tau181 and Other Alzheimer's Disease Biomarkers Identifies Autotaxin–Lysophosphatidic Acid Signaling as Potential Therapeutic Targets

**DOI:** 10.1002/alz70856_107767

**Published:** 2026-01-09

**Authors:** Min N Qiao, Prabesh Bhattarai, Elanur Yilmaz, Alexander W Rookyard, Lipi N Das, Marielba Zerlin‐Esteves, Dolly Reyes‐Dumeyer, Annie J. Lee, Rafael A. Lantigua, Martin Medrano, Diones Rivera Mejia, Lawrence S. Honig, Lewis M Brown, Caghan Kizil, Richard Mayeux, Badri N. Vardarajan

**Affiliations:** ^1^ The Taub Institute for Research on Alzheimer's Disease and the Aging Brain, Columbia University, New York, NY, USA; ^2^ Gertrude H. Sergievsky Center, Columbia University, New York, NY, USA; ^3^ The Taub Institute for Research on Alzheimer's Disease and the Aging Brain, New York, NY, USA; ^4^ Columbia University Irving Medical Center, New York, NY, USA; ^5^ Department of Neurology and The Taub Institute for Research on Alzheimer's Disease and the Aging Brain, Columbia University Irving Medical Center, New York, NY, USA; ^6^ Columbia University, New York, NY, USA; ^7^ Columbia University, Dobbs Ferry, NY, USA; ^8^ Department of Neurology, Columbia University Medical Center, New York, NY, USA; ^9^ Department of Medicine, Vagelos College of Physicians & Surgeons, Columbia University, New York, NY, USA; ^10^ School of Medicine, Pontificia Universidad Catolica Madre y Maestra, Santiago, Santiago, Dominican Republic; ^11^ CEDIMAT, Plaza de la Salud, Santo Domingo, Santo Domingo, Dominican Republic; ^12^ Taub Institute for Research on Alzheimer's Disease and the Aging Brain, Vagelos College of Physicians and Surgeons, Columbia University, New York, NY, USA; ^13^ Department of Neurology, Vagelos College of Physicians and Surgeons, Columbia University, and the New York Presbyterian Hospital, New York, NY, USA; ^14^ Department of Neurology, College of Physicians and Surgeons, Columbia University, New York, NY, USA; ^15^ Departments of Neurology, Psychiatry, and Epidemiology, Gertrude H. Sergievsky Center, The Taub Institute for Research on Alzheimer's Disease and the Aging Brain, Vagelos College of Physicians and Surgeons, Columbia University, New York, NY, USA; ^16^ The Taub Institute for Research on Alzheimer's Disease and the Aging Brain, Vagelos College of Physicians & Surgeons, Columbia University, New York, NY, USA

## Abstract

**Background:**

We investigated the relationship between the cerebrospinal fluid (CSF) proteome in Alzheimer's disease (AD) and the clinical and biomarker‐assisted diagnoses, and with CSF biomarker levels of AD.

**Methods:**

CSF was collected in 500 individuals of non‐Hispanic white, African Americans, and Caribbean Hispanic individuals from Dominican Republic and New York City. CSF biomarkers of AD were measured including *p*‐tau181, Aβ40, Aβ42, total‐tau, neurofilament light chain (NfL) and glial fibrillary acidic protein (GFAP). CSF was depleted of abundant proteins followed by precipitation, cysteine reduction/alkylation, and proteolytic cleavage by trypsin. Peptides were measured using a Q Exactive HF mass spectrometer (Thermo Scientific). Association of individual and co‐abundant modules of proteins were tested with the clinical diagnosis of AD, as well as biologically defined AD pathological process based on CSF *p*‐tau181 and other biomarker levels. Results from replicated in 397 participants from the Accelerated Medicine Partnership‐Alzheimer's Disease CSF cohort and significantly associated proteins were functionally validated in postmortem human brains and zebrafish models.

**Results:**

CSF levels of 41 proteins were significantly associated with *p*‐tau181 levels after multiple testing correction. Notably phospholipase D3 (PLD3, *p* = 2.41E‐09), APOE (*p* = 4.25e‐08) and osteopontin (OSTP, *p* = 1.4E‐16) were increased and autotaxin (ENPP2, *p* =  8.39E‐09) and ceruloplasmin (CERU, *p* = 2.72E‐07) were decreased among individuals with high *p*‐tau181 levels. These proteins were also associated with CSF Aβ42/Aβ40 ratio and total Tau levels but not with NfL. OSTP was also associated with CSF levels of GFAP (*p* = 1.32e‐05). We did not identify any protein association with clinical AD. Among proteins associated with *p*‐tau181 levels, pathways related to axon development (*p* = 2.4E‐12), axonogenesis (*p* = 1.45E‐11) and regulation of axonogenesis (*p* = 5.1E‐09) were enriched. Immunostaining on postmortem human and zebrafish brains found that ENPP2 expression reduces significantly with AD and amyloidosis, respectively. LPA administration into the zebrafish CSF mitigated Aβ42‐induced vascular, neural, and glial changes.

**Conclusion:**

Unbiased profiling of circulating CSF proteins identified key proteins associated with β‐amyloid and phosphorylated tau pathology. Biologically based diagnostic criteria may aid in the identification of unique pathogenic mechanisms.

